# *Cynefin* as Reference Framework to Facilitate Insight and Decision-Making in Complex Contexts of Biomedical Research

**DOI:** 10.3389/fnins.2017.00634

**Published:** 2017-11-14

**Authors:** Gerd Kempermann

**Affiliations:** ^1^German Center for Neurodegenerative Diseases (DZNE) Dresden, Dresden, Germany; ^2^Center for Regenerative Therapies Dresden (CRTD), Technische Universität Dresden, Dresden, Germany

**Keywords:** complexity, decision making, neurodegeneration, management, systems biology, systems medicine

## Abstract

The Cynefin scheme is a concept of knowledge management, originally devised to support decision making in management, but more generally applicable to situations, in which complexity challenges the quality of insight, prediction, and decision. Despite the fact that life itself, and especially the brain and its diseases, are complex to the extent that complexity could be considered their cardinal feature, complex problems in biomedicine are often treated as if they were actually not more than the complicated sum of solvable sub-problems. Because of the emergent properties of complex contexts this is not correct. With a set of clear criteria Cynefin helps to set apart complex problems from “simple/obvious,” “complicated,” “chaotic,” and “disordered” contexts in order to avoid misinterpreting the relevant causality structures. The distinction comes with the insight, which specific kind of knowledge is possible in each of these categories and what are the consequences for resulting decisions and actions. From student's theses over the publication and grant writing process to research politics, misinterpretation of complexity can have problematic or even dangerous consequences, especially in clinical contexts. Conceptualization of problems within a straightforward reference language like Cynefin improves clarity and stringency within projects and facilitates communication and decision-making about them.

## Introduction

We regard many, if not most important biological and medical questions as complex, because they do not have single straightforward solutions. Life itself is complex and is more than the sum of its part: simple, direct causalities are only found within defined functional modules, such as chemical reactions and physical interactions (and even there can be challenged). Insight on larger-scale questions about “life” cannot be fully reconstructed bottom-up only from such modules. In large networks, probabilistic relationships and highly indirect influences blur mechanistic insight. Complex problems and questions in the life sciences have “*emerging properties*” that preclude that there are single true answers to questions of causalities. Cancer, neurodegeneration, consciousness, etc., cannot be comprehensively understood simply by adding up insights from partial aspects. They require a more holistic perspective, which, however, might ultimately be impossible to gain. While the large questions of life are the most obvious manifestations of a problem with complexity, the issue actually permeates biomedical research at every level.

In the clinical context this is much more obvious than in basic research. If an average 75 year-old patient accumulates five or more medical diagnoses, which all might lead to a more or less specific treatment, the resulting matrix of past, present, and future interactions at the level of the development of the disease, the co-developing (intrinsic) strategies of coping with the problems and the, in most cases: pharmacological, (extrinsic) treatments amount to a high-dimensional complexity of causalities and interferences that cannot be grasped–especially not intuitively in the emergency room. Even if these networks could be modeled theoretically, this will be of limited value in the acute situation. Medicine for older people is, even more than for younger subjects, a medicine of complexity and so is the underlying biology. The reason that many, especially chronic, diseases have not yet been conquered lies in exactly their complexity and our imperfect grip on this complexity.

The problem is, however, neither limited to aged subjects or humans, nor to medicine. Biomedical research as the science of life is interested in causes and mechanisms underlying complex *quantitative* processes. Phenotypes tend to be gradual and are often found in a normal distribution. The strengths of reductionism generally turn into a risk, when the key to the sought solution lies in the emergent properties, which hide in the observable and measurable phenotypic appearance of complex traits, behaviors, diseases, etc., Most human traits, including the susceptibility to disease, are highly polygenic and to a variable degree subject to environmental and behavioral influences. The co-development of physiological traits with pathology results in extreme multi-dimensional complexity, challenging our ability to understand appropriately and act accordingly. In principle, the problem is thus relevant to any combination of physiological (and pathological) traits and across scales. This most elusive aspect of the picture might be the most critical one, especially for example, if it comes to translation from models to the clinic. Controlling for complexity must not result in ignoring it. The interpretation of the results might fail, if the full context is not appreciated, even though we might not be able to fully comprehend it and factor it in. So the first step is to be clearer in language and conceptualization.

While the diagnosis of complexity has thus become ubiquitous, concrete systems approaches in biology and medicine are attempts to extract usable insight from this complexity in order to guide novel approaches to gain insight on one side for treatment and for prevention on the other. With “systems biology,” “systems genetics,” “systems medicine” etc., sub-disciplines have evolved that specifically and by definition address the complexity of life, but the very same challenges permeate everyday research and medicine much more than is often realized. Quantitative biology calls for a particular mindset that is not always appreciated outside the seemingly fenced-off “systems” fields. In reality, however, essentially all biology is “systems,” because all biology deals with life and essentially all of biology has become quantitative. A better way to integrate “systems” ideas into normal biology and medicine is thus necessary.

In addition, outside the inner, concrete medical or clinical context the desire for insight into complex biological causalities is interwoven with a variable degree of translational intentions and aims, adding new layers of interdependencies and influences. Across the highly diverse spectrum of stakeholders from academic science and education over industrial research and development with its economic perspective, to societal and political interest groups, including patients, there is a wide range of perceptions of how our answers to complex questions and problems in biomedicine should look. Interpretation and decisions are not pure. The construct of science in our society is “multirational,” which means that overall aims, strategies, approaches, etc., do not follow one single rationale, but might be ill-aligned or conflicting. The perspective of a zebrafish or mouse researcher on “Alzheimer” is different from a clinician or a care professional, who nominally acts in the same field.

In this situation, it is an additional problem that biology as a whole has been relatively theory-adverse in coming to terms with what, in such situation, might constitute “mechanisms” and how we can interpret them. There are of course activities toward this end [for example in the work by Lindley Darden (Craver and Darden, 2013)], but appreciation has not generally caught on that a constant reflection on implicit assumptions and standards as well as the fundamental properties of the subjects are required in order to be successful.

It is also important to realize that scientific culture matters. Focus on the inner circle of biological reasoning has possibly prevented us from realizing that with “Cynefin” a relatively straightforward tool has been around for almost two decades which might help to change this situation and which we should now discover for the life sciences.

## The Cynefin framework

The Cynefin framework has been a influential concepts in management but largely unknown in the natural sciences and medicine. Cynefin was first published in 2000 by Snowden (2000). A later paper in the Harvard Business Review from 2007 provided a first generalization as strategy model irradiating well beyond the original management contexts (Snowden and Boone, 2007). Cynefin (*kuh-ne-vin*) is the welsh word for habitat or rootedness and was used by Snowden as metaphor for a conceptual framework for “time and place” to make decisions in complex situations. Complexity is certainly no privilege to business and economy, however, and the arguments that Snowden and others developed generalize easily to other fields. It has, for example, been famously used to describe (and make sense of) the decision making during the G. W. Bush presidency (ONeill, 2004). An application to later administrations might be similarly enlightening.

There have also been some applications to medicine (Mark, 2006; Sturmberg and Martin, 2008; Van Beurden et al., 2013) but by and large, Cynefin has not yet had a measurable impact on life sciences. This might have cultural reasons and be partly due to the emphasis on decision making that Snowden's original text had, but the tools from Cynefin to make sense of complexity appear to be highly useful for current biology and medicine as well.

**Figure 1:**
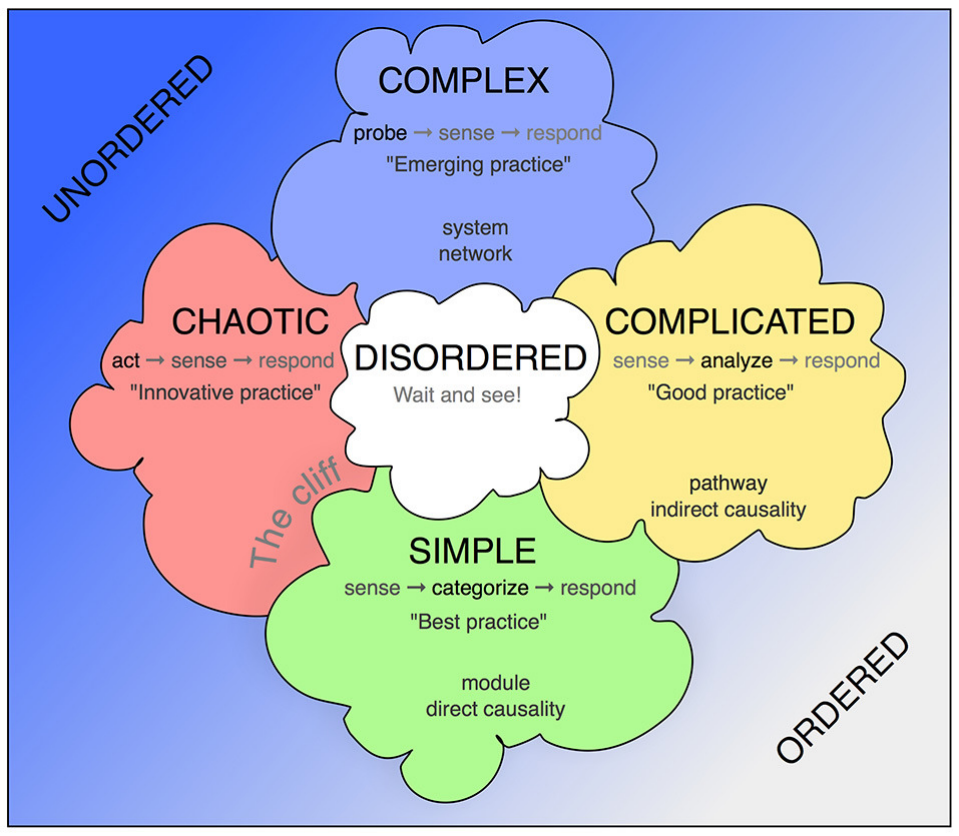
The Cynefin scheme. This version is one of the many renderings of the Cynefin framework highlighting and explaining the five core contexts. Unlike some other replications, this version uses the arrangement from Snowden's original publication from 2007, in which the square stands on one corner, so that the “simple/obvious” category is found at the base and the “complex” category at the top. More commonly found is an arrangement with “chaotic” and “simple” next to each other at the bottom and “complex” and “complicated” as a top row. The version here has the advantage of highlighting the complex contents and assigning the simple contexts in their modularity a role at the basis. In some fields, quintessential concepts from biomedicine have been added.

The Cynefin framework allows contextualization of situations that require a decision and response by providing a reference language (Figure 1). The key assumption is that such situations fall into one of five categories, which call for substantially different but definable conclusions and mode of actions, and that awareness of these principal differences allows more structured insight and better-informed decisions.

## Simple or obvious contexts

These are the domain of clear-cut causality, in which B follows from A. Dealing with simple or obvious contexts requires to describe (“sense”) the situation, assign it to pre-established categories and act according to the defined procedure for that category. The resulting action can adhere to fixed routines, called “best practice” or standard operating procedures. Simple context are the procedural backbone of more complex contexts. In the language of systems biology they relate to the modules, self-contained processes and functional units, such as protein-protein interactions, basic chemical reactions, etc.

In research, simple contexts comprise only what is known already: the “known knowns.” But what is known does not raise questions anymore and without questions no science exists. Simple contexts are thus only the building blocks for addressing the larger questions. Standard operating procedures can invariably apply only to methods that are used, not the entire experiment. Mistaking questions as simple and thus applying only the SOPs will almost invariably miss the point.

A single mutation, for example, does not generate a simple context. The knockout of any gene has reverberations throughout the genome that go far beyond the immediate function of that gene. Relatedly, the same mutation has different consequences on different genetic backgrounds (Sittig et al., 2016). Even classical Mendelian genetics is not simple or obvious, and monogenic does not mean mono-causal.

## Complicated contexts

Contexts, in which causalities can at least in theory (or retrospect) be known but are non-linear and difficult to untangle are called “complicated.” In this category the causalities are potentially discoverable and this is why at this level the first research questions can appear. We here deal with “unknown knowns.” which means that the questions can be addressed by not venturing too far from what is established, often in a way of extrapolation or combination, sideways application or, to some degree, generalization.

Complicated contexts have no single, obvious best solution and thus are the domain of experts (and consortia). Hence not best but only “good practice.” is possible. In research complicated contexts are common, but they are not necessarily the most important and interesting contexts, because they ultimately do not leave the comfort zone in terms of the openness of the possible question. Complicated problems can certainly be *very* complicated and pose large organizational and other challenges. Many cohort studies, for example, would initially fall into this category of massive undertakings, that while addressing complex questions, by design are “only” complicated. The distinction and its consequences are critical. Only once the data starts becoming available the efforts to untangle complexity within it can be started. This level has a different structure than the organization of the data acquisition. The genome project is an example of such project, in which the highly complicated endeavor led to a excessively complex data set.

If seen from the biological fact not the technological achievement, one would say that, conversely to common perception, complex situations might require approximations by means of complicated tools, whose inherent limitations have to be recognized and factored into the interpretation.

Boyle, Li and Pritchard have argued that for complex traits, the classical description as “polygenic” is not fully appropriate because the underlying causal networks extend to essentially the entire genome with minimal effect sizes from most genes, which in their entirety nevertheless explain most of the variance. They have coined the term “omnigenic” for this relationship (Boyle et al., 2017).

In addition, much of the more classical exploratory research (expeditions, field studies, etc.,) will be found in this category, but, again, not necessarily their results and the ensuing next questions. A consequential mistake would arise from the failure to realize that more than complicated research this is needed to address complexity.

## Complex contexts

Although it is only one of the five contexts described by the Cynefin framework, complexity actually lies at the heart of the idea. The entire framework has been designed in order to identify this critical category correctly and allow decisions (actions) that are appropriate to the specific demands of these contexts, also in relation to the others. The complex contexts are those of the “known unknowns,” in which neither “best” nor “good” practice can be used, but “emergent practice” is needed. This description alone already suggests, why for the research context this category is fundamental.

As stated above, “complex” and “complicated” is not the same. In complex contexts we face a situation, in which key aspects are non-observable, latent or emergent. Here, (as well as the chaotic situation described next) action has to precede proper apprehension, in complex contexts in the form of probing the context. The complex situation is such that it has to be probed through (experimental) perturbations in order to reveal enough of the underlying causality structure to allow proper diagnosis and consequently insight and decision. That action is required before insight is possible is, fundamentally different from simple/obvious or complicated contexts, where a certain level understanding can or even must precede action. Higher-order biological functions or diseases like neurodegeneration or cancer obviously have these complex properties but the feature pervades seemingly much simpler contexts. Reducing complex processes of life, for example, to the addition of single-gene effects and accepting descriptive changes in individual signaling pathways (e.g., through KEGG) as sufficient approximation to complexity and as convincing “mechanism” might be deceiving and result in false explanations and simplifications (van Swinderen, 2005). The same is true, if we blindly assume that causalities uniformly act across scales, from molecules to behavioral and social. And finally, the same macroscopic outcome can be achieved through multiple, different mechanisms (Marder, 2011).

**Figure 2:**
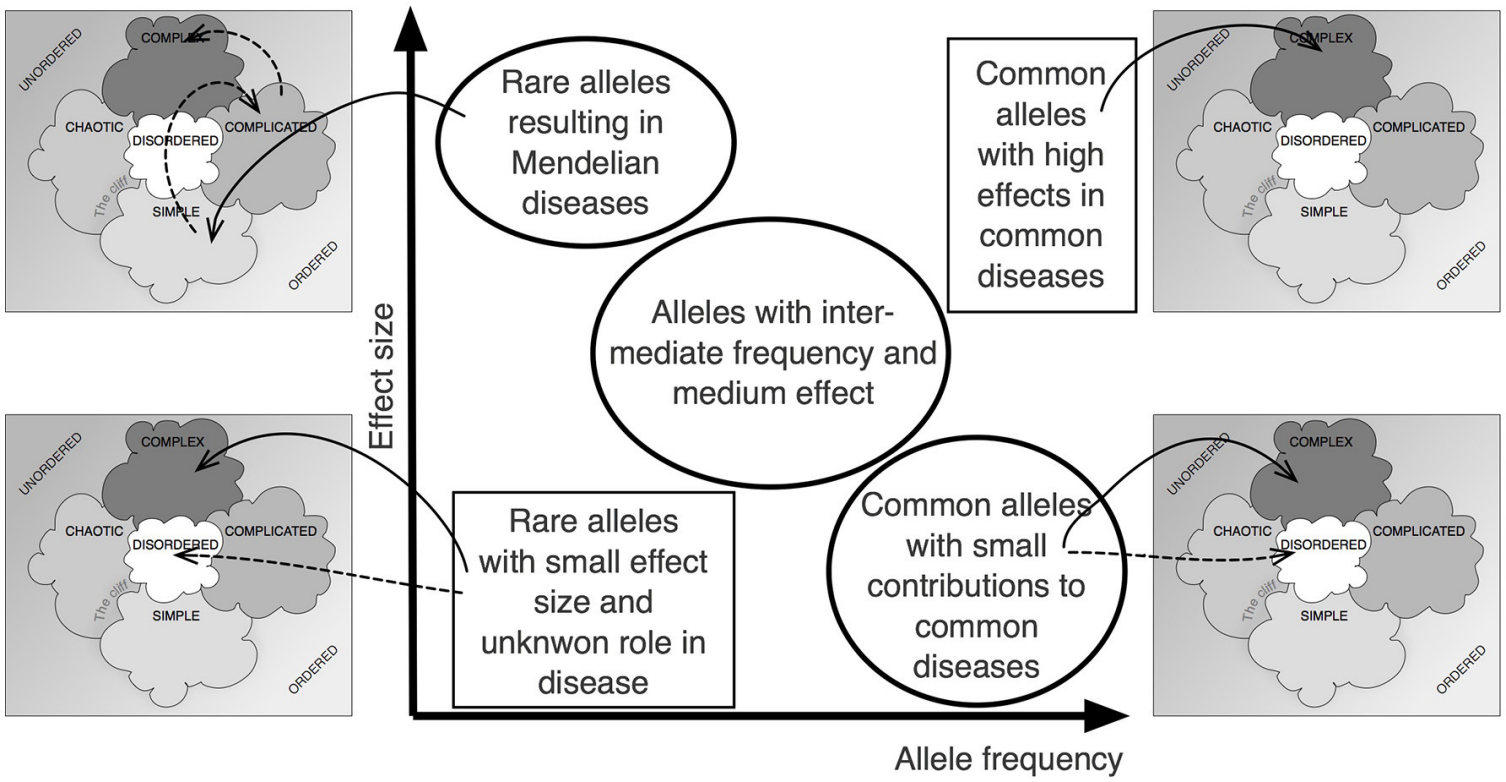
The Cynefin scheme in complex genetics. Plotting effect size over allele frequency has been an important approach to visualize the complex situation underlying the noted “missing heritability,” the difference between overall heritability and the additive effect of identifiable gene loci (Manolio et al., 2009). Applying the Cynefin scheme to this pattern highlights the fact that contexts differ vastly across that scheme and that, thus, different consequences must arise. The statement that the situation is “complex” becomes enriched by details.

Figure 2 shows how the Cynefin framework can be applied to the central relationship between allele frequency and effect size in complex, polygenic traits (Manolio et al., 2009). It becomes obvious that different positions within the relationship depicted by that graph require very different approaches, decisions and actions. In some sense, the various fields of the graph are not comparable. It is not simple or even only complicated to close the gap between the calculated heritability of a trait and the total effect size of the known genetic variants (“missing heritability”).

## Chaotic contexts

Biological systems can be chaotic for various reasons: massive acute or late-stage disease, such as cancer are examples of chaotic breakdown of formerly orderly systems, but false security in dealing with biomedical phenomena are another, more subtle source of chaotic biomedical systems.

In the first cases, the reasons for chaos are immanent, in the seconds extrinsic or technical.

Many biological systems are said to be “poised at equilibrium,” which is often compared to a tight-rope walk and means that they border to chaos and fall (Mora and Bialek, 2011). Biological systems tend to push the extremes, and the resulting equilibria are consequently fragile. If they break down, the picture does not allow conclusions about the previous, orderly state and about the rules that governed it. Correctly diagnosing chaos and distinguishing it from complexity is crucial for understanding biological systems. A single-gene knockout might have reverberating consequences for large genetic networks, so that despite the defined molecular lesion the consequences become unintelligible. Despite being seemingly “simple” no assessable cause-consequence relationship remains detectable at the systems level. The mutation's effects dissolve in a flurry of loosely interacting changes across the system, often manifesting itself in high variance. But in many experimental settings, chaotic conditions might actually be hidden.

Complexity has to be distinguished from chaos because both require different actions. In chaotic systems, like in complex contexts, action is required, but in chaos this action first consists of crisis intervention to steer the system into calmer waters. This intervention goes beyond probing and, importantly, is not targeting insight and decision making but is an emergency measure for stabilization.

Practical medicine might run much greater danger than pure research to face chaotic situations that result in conditions which are much closer to the leadership issues that Snowden described in his original publication. An acute ischemic stroke, for example, calls for lysis treatment within the shortest possible time; all further diagnosis and therapy must wait.

Nevertheless, there have been only few direct applications of the Cynefin framework to Medicine. Pubmed as the main publication data base in this area currently (September 2017) lists a mere 10 reports with the keyword, mostly related to public health issues, not research or clinical practice. And none to decision making in medical emergencies.

In research, chaotically failing experiments might have to be abandoned with less dire consequences than the death of a patient but structurally, the problem is similar.

Chaotic contexts require “innovative practice” and cannot be dealt with the common toolbox. Note that the intervention might or must nevertheless follow simple protocols and that even the next steps might by necessity apply merely complicated routines, failing to address the underlying complexity. According to the logic of Cynefin, it thus becomes evident, in which respect such solutions are limited. The reference framework has the purpose to point to such critical limitations (as well as to the opportunities).

## Disordered contexts

A key insight by Snowden and colleagues has been that many contexts cannot be categorized immediately and require a “wait and see” approach in order to appreciate into which of the other four causality categories they would fall. Especially complex contexts have a propensity to immanent change and development.

In the scientific context the “disordered” category is particularly interesting, because it includes many white spots on the map of knowledge. Being alert, sensitive and patient to recognize windows of opportunities in contexts that are not yet ready for mainstream creates opportunities. Applying Cynefin helps identifying contexts, which are worth further observation and careful probing. This does not implicate that the frontiers of knowledge necessarily have to present themselves as “disordered,” but only that “disordered” might be an indicator of novelty. The category will also include ballast, observations at the limits of technical resolution or plainly erroneous material (in which “wait and see” will ultimately not be fruitful).

In complex genetics, for example, common polymorphisms with small effect sizes test the limits of resolution and require distinction from mere stochasticity and noise in the system, before any incorporation into complex models of polygenic causality is possible.

## The cliff

The boundary between “simple” and “chaotic” contexts, in schematic drawings of the Cynefin framework often rendered as cliff, is dangerous, because a categorization as “simple” or “obvious” might lead to false security and premature conclusions. Because a straightforward causality is mistakenly assumed, the conclusions and resulting actions are inappropriate, leading to chaos. The context changes from seemingly simple to chaotic. In medicine this might relate to missing the chance for the appropriate surgical intervention because under an incorrect causality model symptoms have been treated too long with inadequate alternative medicine. In fundamental research similar constellations might underlie much of the irreproducibility crisis or whenever overly simplistic models of disease lead to the identification of pharmacological targets that fail to live up to the expectations in following clinical applications. A key insight from the Cynefin framework is that there is often no easy way back from the resulting chaotic conditions to simpler contexts.

There is a tendency in research (as in management as one learns) to move all phenomena to the complicated and simple fields, rather than to complexity, where, in fact, most issues will be and remain at home. Incorrectly identifying a context as complex, when it is only complicated, has little damaging consequence (except for the “impact” of the resulting paper), whereas missing the complexity of a context and mislabeling it as only complicated (or even simple) might have dire consequences. It is better to err on the more demanding side and re-categorize downwards than to “fall from the cliff.” The worst scenario would be the failure to recognize that this fall has happened.

In a small departure from the original Cynefin design one might with reference to biomedical research be inclined to extend the cliff also a distance into the complicated contexts.

## Complex biomedical problems have aspects that are complicated or even simple/obvious

While most biomedical questions are complex, most biomedical problems have parts that are complicated or simple. This implies that to a certain extent, an action according to these categories will be appropriate. In Parkinson's disease (PD), treatment with dopamine precursor levodopa (L-dopa) usually is the initial therapy of choice and for basic diagnostics guidelines or SOPs can be developed at this level (Berardelli et al., 2013; Martí and Tolosa, 2013). At a later stage of the disease, to rely only on these SOPs will miserably fail and an extensive individual fine-tuning will become necessary. In systems biology, such basic, relatively self-contained units are called “modules” and although the concepts are not entirely congruent, to think of identifiable simple or moderately complicated relationships within complex contexts as modules will often be helpful and informative. But the disease (or other scientific problem) is more than the sum of the modules and any module points outside of itself. From a practical point of view, to break down complexity into manageable units is tempting and even necessary. But this approach might lead to the implicit belief that by working off the list of modules, the larger question can be answered. However, no additive bottom-up strategy can be successful in answering complex questions. This lies in the particular emergent properties of complex contexts. What is needed is a framework to make best use of modules to capture the complexity of their context. In research, individual classical experiments are such modules. But many publications fall short of highlighting (to say nothing of explaining) the gap between the sum of the experiments and their results and the greater answer that is actually sought. Raising awareness of the general properties of complex contexts, for example within a framework, such as Cynefin, can improve judgment and decision, put insight into perspective, facilitate realistic concrete translation and reduce the risk that complexity is simply ignored.

## Decision making in research

In contrast to management, where Cynefin came from, biomedical research usually does not overtly emphasize decision making. This is different in the purely clinical setting, but a potentially damaging misconception lies in the implicit idea that in most other biomedical contexts decision making would not be a key issue. But this is not correct. While there are parts of medicine that are closer to management of the kind represented by “*Harvard Business Review*” than research (for example administering a hospital or managing a large clinical study), science itself is in fact crucially dependent on decision making and a particular kind of management as well. But this is rarely made explicit and is often left to intuition or neglected as soft factor with marginal impact. But research is action, organization, communication, and leadership as much as it is creativity, knowledge, persistence and luck. There is also a “market” for research results, spanning out between grant writing, collaborating, publishing and lobbying that sets the stage, on which researchers from their Ph.D. level onwards have to act. There is no such thing as pure science, and science does not naturally self-evolve. Science is led and managed, and whether we like it or not, science has consistently to be “sold” at every level. This is no digression from the elysian, innocent conditions in an ideal past but a fundamental property, because science itself is a complex human endeavor.

Every experiment requires decisions during every single phase. When experiments (and the questions they attempt to answer) are simple, this is no issue. The staged “experiment” that the physics teacher performs to demonstrate *F* = *m* × *a* is predictable down to the boredom of the pupils. Such experiments are replicative and “best practice,” not set up to push the borders of knowledge in general, but only in the audience. The experiments, as any application of “best practice,” are not used to inform larger decisions.

If a diabetic patient routinely measures blood glucose to decide on the required dose of insulin in the next injection, the underlying causality context is “simple.” Nevertheless, the same context is complex from a scientific perspective, resulting in different options and resulting decision-making processes. The practical level differs profoundly from the scientific level. And despite standard operating procedures of insulin measurement and injections schemes, this approach might fail, because the underlying complex reality cannot be captured with this approach.

As most real and novel experimental contexts are complicated or complex, various levels of management and decision making are necessary to initiate the research, perform it, interpret it, communicate it and thereby make it successful. This starts, of course, with the implicit and explicit decisions on the right questions.

In complex research contexts with their emergent properties causality cannot be fully predicted a priori but only approximated to guide the experimental process. The stringency of the hypotheses varies. Iterative processes with computational modeling and experimental verification lie at the heart of any “systems” approach as the accepted answer to biological and medical complexity. Initial questions here tend to be extremely open, so that they do not qualify as “hypothesis” anymore, although this might be a semantic issue and the decision lie in the eyes of the beholder. As a consequence, the answers deduced from the results of such research might be more concrete than the question that has originally been asked, invariably leading to a retrograde, reconstruction of a “storyline” that satisfies our desire for simplicity and a narrative structure. From a communication point of view this might be justifiable and even necessary, but it would require a wider appreciation that such reduced stories are punchlines of a generally complex world in order to avoid larger scale misunderstandings and inflated hopes for translation. Reductionism, simplification, narrative structures, etc., will always be necessary but need to be seen in the correct context of the real complexity (or chaos or disorderedness).

The Cynefin framework is one way of sensitizing at least the researchers, grant agencies, journalists and politicians (as the mainly involved managers) to the the complexity in biomedical research and its consequences to insight and decision making.

“Case manager” are an established solution to addressing (and reducing) clinical complexity in cancer, dementia and neurodegeneration and arrive at executable best solutions (Huston, 2002). The Cynefin scheme provides a straightforward framework to convey the necessity and benefits of such solutions to all stakeholders. Multi-disciplinary case management in science is still rare.

## Case study: Parkinson's disease

Parkinson's disease (PD) is caused by the degeneration of dopaminergic neurons in the substantia nigra of the midbrain resulting in a characteristic pattern of motoric symptoms. This is a “simple,” monocausal description of the disease that is widely found in publications and is about the level of knowledge even of many health professionals. The obvious treatment option according to this description has been the extrinsic replacement of dopamine in the form of L-dopa, which indeed has the power of relieving the symptoms. But generally not quite. Some symptoms respond better than others, the effect might fluctuate over time and it wears off (Salat and Tolosa, 2013). There is also a massive inter-individual variation in the response. Treatment with L-dopa is initially the approach of choice in most patients, but over time inherent limits become apparent.

But there is a large variation in the clinical picture and the genetic causes anyway (Riess et al., 2000; Mullin and Schapira, 2015). The classical triade of tremor, rigor, and akinesia is found to a variable degree, there are non-motor symptoms, including dementia, the disease has a long clinically silent latency period, during which conditions, such as certain sleep disturbances might show up as first indicators and there are mixed forms with other types of neurodegeneration (Berardelli et al., 2013; Martí and Tolosa, 2013). Such situation obviously qualifies as “complicated” at the very least. Treatment requires expert knowledge in weighing symptoms and progression against treatment options. There is generally no single correct solution that fits all situations. Therapy belongs, at least temporally, into the hands of specialists, who can collect that information. We are at the upper-right quadrant of the Cynefin scheme.

But the disease is actually complex and while treating it as “complicated” is better than simplifying it, still has serious limitations. The genetics of PD reveal that the sporadic forms of the disease are polygenic with low effect sizes for individual loci (Riess et al., 2000; Mullin and Schapira, 2015). Most of these do not show any overt relationship to the dopaminergic system. The main pathogenic mechanism relates to the accumulation of alpha-synuclein, but mutations related to the alpha-synuclein gene account for only a very small subset of the disease (Lashuel et al., 2013). Polymorphisms in the Lrkk2 gene shows a high association with sporadic PD, but the effects are highly variable and no single mechanism has as yet emerged, how Lrkk2 might be causally involved in PD. It seems that no such single, unique relationship exists (Esteves et al., 2014).

In addition, environmental factors obviously play an important role, ranging from toxins, especially pesticides, to lifestyle risk and resilience factors (Checkoway and Nelson, 1999). The relative contribution and interaction of all of these is not really known and no comprehensive model of the disease exists. By all standards, PD qualifies as “complex” with all the consequences for insight and decision making. Sticking with a “simple” disease concept is prone to failure, with all the consequences of “falling over the cliff.” Concretely, the patient might be overwhelmed by side-effects, might not be treated for the full range of symptoms, and critical differential diagnoses are missed. Appreciating that the situation is complicated, in contrast, still misses the critical point that there are aspects which cannot be known in complex contexts and, most importantly, that finding solutions are approximations.

## Organizational consequences

The original Cynefin framework emphasizes the social and cultural context. The approach has been designed to elucidate the relationship between individual, experience and context. The generalization to a tool of knowledge management allows the application to science and research at the level of contents, as outlined in this article, not just organization. But there are of course opportunities for combinations of the different domains, insight and knowledge on one side and decision-making and organization on the other. In fact they are closely related and will constantly inform and influence each other.

Some life-scientists might at first tend to find Cynefin rather common-sensical, but what they criticize in the concept might actually be an advantage. First in the sense of Ockham's razor, that the more simple description might actually the better one, but also by facilitating acceptance on the long run. Snowden characterized his tool as one whose outcome could be sketched on a napkin.

The point is not to invent a new layer of complexity that has to be dealt with in addition to the complexity that prevails anyway but to develop a culture in biomedical research that appreciates and accepts complexity and provides a reference framework and common language.

Many scientists experience the current organization of science and research as increasingly inadequate. Research in the life sciences is becoming more and more differentiated and specialized and takes place under an increasing numbers of preconditions that we are usually not aware of. These conditions includes opportunities and constraints and require additional efforts to decipher their impact and act accordingly.

## Conclusions

The reproducibility crisis, the conceptual and practical challenges of multi-omics and big data, the discussions around personalized medicine, the heritability gap in genome wide association studies, the failure of clinical trials for cancer and neurodegeneration after successful animal experiments and the tremendous difficulties in defining criteria for satisfying “mechanistic” explanations of a biological observation are all examples of struggles with complexity in biomedicine. Living organism are complex, because they are adaptive, dynamic, self-organizing, developing, learning, etc., and have emergent properties, but in most scientific projects, from design to publication, and from Ph.D. theses over project reviews to institutional evaluations and research politics, consequences of complexity on results, insight and decisions are not considered systematically. The Cynefin scheme, originally published as tool for decision-making in management has developed into a broadly applicable approach for knowledge management, but remains to be discovered also by biologists and clinical researchers to conceptually and practically deal with complexity. Cynefin is no substitute for concrete scientific solutions to the consequences of complexity, but provides a common reference language and conceptual framework to talk about complexity and draw the appropriate conclusions for insight, decisions and actions. For biomedical research in general, Cynefin helps to capture the critical consequences of complexity at the place, where they arise, and prevents their sequestration into the specialist domains of “systems” biomedicine.

## Author contributions

The author confirms being the sole contributor of this work and approved it for publication.

### Conflict of interest statement

The author declares that the research was conducted in the absence of any commercial or financial relationships that could be construed as a potential conflict of interest.
